# The epidemiology of chronic pain in Libya: a cross-sectional telephone survey

**DOI:** 10.1186/s12889-016-3349-6

**Published:** 2016-08-11

**Authors:** Raga A. Elzahaf, Mark I. Johnson, Osama A. Tashani

**Affiliations:** 1 Centre for Pain Research, Faculty of Health and Social Sciences, Leeds Beckett University, Leeds, UK; 2 Leeds Pallium Research Group, Leeds, UK; 3 Faculty of Medical Technology, Derna, Libya; 4 University of Benghazi, Benghazi, Libya

**Keywords:** Pain, Epidemiology, Prevalence, Chronic pain, Libya, Middle East and North Africa (MENA), Developing world

## Abstract

**Background:**

Chronic pain is a public health problem although there is a paucity of prevalence data from countries in the Middle East and North Africa. The aim of this study was to estimate the prevalence of chronic pain and neuropathic pain in a sample of the general adult population in Libya.

**Methods:**

A cross-sectional telephone survey was conducted before the onset of the Libyan Civil War (February 2011) on a sample of self-declared Libyans who had a landline telephone and were at least 18 years of age. Random sampling of household telephone number dialling was undertaken in three major cities and interviews conducted using an Arabic version of the Structured Telephone Interviews Questionnaire on Chronic Pain previously used to collect data in Europe. In addition, an Arabic version of S-LANSS was used. 1212 individuals were interviewed (response rate = 95.1 %, mean age = 37.8 ± 13.9 years, female = 54.6 %).

**Results:**

The prevalence of chronic pain ≥ 3 months was 19.6 % (95 % CI 14.6 % to 24.6 %) with a mean ± SD duration of pain of 6 · 5 ± 5 · 7 years and a higher prevalence for women. The prevalence of neuropathic pain in the respondents reporting chronic pain was 19 · 7 % (95 % CI 14 · 6-24 · 7), equivalent to 3 · 9 % (95 % CI 2 · 8 to 5 · 0 %) of the general adult population. Only, 71 (29 · 8 %) of respondents reported that their pain was being adequately controlled.

**Conclusions:**

The prevalence of chronic pain in the general adult population of Libya was approximately 20 % and comparable with Europe and North America. This suggests that chronic pain is a public health problem in Libya. Risk factors are being a woman, advanced age and unemployment. There is a need for improved health policies in Libya to ensure that patients with chronic pain receive effective management.

## Background

Chronic pain is a global public health concern because of its high prevalence, high economic costs, and negative impact on the quality of life of individuals and their families. It is claimed that the impact of chronic pain on individuals and the burden of chronic pain on societies is similar across the world, although the prevalence and determinants of chronic pain may vary within and between regions because of differences in standards of living, healthcare resources and the high prevalence of pain-generating diseases [1]. There has been limited research on the epidemiology of pain in countries of the Middle East and North Africa (MENA). Research to date suggests that there is likely to be a high prevalence of conditions that contribute to neuropathic pain including HIV/AIDs, diabetes mellitus, cancer and traumatic injuries associated with accidents and on-going conflicts [2–5]. In 2010 an expert panel claimed that much neuropathic pain remained underdiagnosed in the Middle East and North Africa [6].

Most Middle East and North African countries are categorised as ‘developing’ with Human Development Indices (HDI) from 0 · 576 in Yemen to 0 · 935 in Israel. The HDI is a measure of the impact of economic policies on quality of life and reflects a nation’s health and longevity, education and living standards. Recently, we conducted a systematic review that found no correlation between HDI and the prevalence of chronic pain [7]. Prevalence in countries with a HDI <0 · 9 was 33 · 9 % ± 14 · 5 % and significantly higher than prevalence in countries with a HDI ≥0 · 9 (29 · 9 % ± 12 · 7 %). Our analysis was undermined by heterogeneity in study methodologies and contamination of samples by comorbidities resulting in large variation in estimates of prevalence by different investigating teams. Most of the data for countries with an HDI < 0 · 9 was generated from 3 multinational epidemiological studies [8–10] with no studies that focussed on a country with a HDI <0 · 9 being eligible for review.

Previously, we have translated the Arabic version of the Structured Telephone Interviews Questionnaire on Chronic Pain that had been used in the Pain in Europe survey [10] and demonstrated its reliability and linguistic validity for use in a Libyan population [11]. In February 2010, we used the Arabic version of the Structured Telephone Interviews Questionnaire on Chronic Pain in a pilot study in the city of Derna, Libya that estimated prevalence of chronic pain ≥3 months to be 25 · 0 % (95 % CI, 16 · 7 % to 33 · 3 %)[10]. We also found that 50 · 0 % (95 % CI: 30 · 8 % to 69 · 2) of the respondents who reported chronic pain scored ≥12 on the Arabic version of the S-LANSS suggesting that their pain was predominantly neuropathic in origin [11]. It is important to confirm these estimates generated from a small sample size. A telephone survey that addresses methodological issues arising from our pilot study is needed.

In Libya, pain management is not a high priority. The availability of opioid medication is limited and pain-related government policies are absent. The aim of the present study was to estimate the prevalence of chronic pain in Libya with particular reference to neuropathic pain. The study was designed to evaluate the relationship between the chronic pain and socio-demographic factors; to describe the pain characteristics among people who suffer from chronic pain; and to gather information on aetiology, diagnosis, severity, duration, impact on quality of life, treatments and attitudes about living with chronic pain.

## Methods

### Study design

A cross-sectional telephone interview survey approach was used to gather data from 1 June 2010 to 3 September 2010, before the onset of the Libyan conflict in February 2011. The study was approved by the Research Ethics Committees of Leeds Metropolitan University (now Leeds Beckett University), Leeds, UK and the Faculty of Medical Technology, Derna, Libya.

Methodology was developed following the results of pilot studies that had translated the following questionnaires into Arabic and validated their use for telephone interviews in Libyan populations [11–13]:

- The Structured Telephone Interviews Questionnaire on Chronic Pain which was used to collect the data for the Pain in Europe survey in 2006 [10]. This questionnaire was used to estimate the prevalence of chronic pain of ≥3 months duration and to gather information about aetiology, treatments and attitudes about living with chronic pain [12];
- The Self-completed Leeds Assessment of Neuropathic Symptoms and Signs (S-LANSS) questionnaire to estimate prevalence of neuropathic pain [11, 13]

### Sample population and size

The most recent census conducted in 2005 found that 97 % of the Libyan population were of Arab or a mixture of Arab and Berber ethnicity. The remaining 3 % of the Libyan population were of Greek, Maltese, Italian, Egyptian, Pakistani, Turkish, Indian, Tunisian and sub-Saharan origin. A sample population that reflected this ethnic mix was obtained by gathering data from the capital cities of the three main regions in Libya. These cities also had the most developed telephone systems. They were:

- Tripoli, the nation’s capital within the Tripolitania region (population = 911,643, predominantly Arabic);
- Benghazi, the capital of Cyrenaica region (population = 685,367, predominantly Arabic);
- Sabha, the capital of Fezzan region (population = 137,307, mixture of Arab and sub-Saharan African Black descent).

Sample size was calculated using data collected from a pilot study in Derna city [12]. Raosoft software (Federal Way, Washington, USA) was used to calculate the number of people required to be screened to detect a 25 % prevalence of pain, with a 3 % margin of error (95 % power at the 5 % significant level). A total of 1212 people were required.

### Data collection and processing

Data was collected using telephone numbers that were randomly selected using computer-generated random numbers (www.random.org) and a random digit telephone dialling system. Any household resident aged 18 and over who answered the telephone was invited to participate in the survey. Respondents were asked if there was more than one adult in the household at the time of the call and if so one of these adults was selected at random.

### Telephone interviews

Telephone calls making the first point of contact with potential participants were made between 10:00 am and 10:00 pm Saturday to Thursday from 1 June 2010 to 3 September 2010. A telephone call was considered unsuccessful if the number did not connect at the time of calling, was connected to a fax machine, or an engaged tone was encountered and/or no one replied after 3 repeat calls at different times on different days. A call was also considered unsuccessful if there was nobody in the house eligible for the study at the time of the call.

Telephone interviews were conducted in Arabic by Libyan nationals using Libyan dialect and following scripts and questionnaires written in Arabic. Interviews consisted of an introduction to study, screening to establish the presence or otherwise of chronic pain, and an in-depth interview consisting of the Structured Telephone Interview Questionnaire on Chronic Pain and S-LANSS (Self-completed Leeds Assessment of Neuropathic Symptoms and Signs). The first point of contact was conducted by the principal investigator (RAE) and two academic staff members from the Faculty of Medical technology in Derna who had received training on telephone interview techniques specific to the study.

#### Screening

The interviewer read verbatim an Arabic translation of the Structured Telephone Interviews Questionnaire on Chronic Pain [10] and transcribed answers onto hard copy. Questions 1–12 were used to determine the prevalence of pain, its cause and treatments used. Respondents were categorised as having chronic pain of ≥3 months according to their answer to question 6 “For how long have you suffered pain due to your illness or medical condition?” The criteria used to determine whether a respondent was categorised as having chronic pain was based on the definition from the International Association for the Study of Pain which uses a cut point of ≥3 months. This differs from that used in the original Structured Telephone Interviews Questionnaire on Chronic Pain which used a cut point of ≥6 months.

Respondents categorised without chronic pain were asked 7 demographic questions after which the interview was terminated. Respondents categorised with chronic pain were asked a further 44 questions about their pain including Question 7 “When was the last time you experienced pain?” which was used to confirm that respondents were correctly categorised as having pain of ≥3 months. Respondents categorised with chronic pain were then invited to take part in the in-depth interview either immediately or on an alternative date. If they declined the invitation for an in-depth-interview they were asked 7 demographic questions (Q38-44) and the interview was terminated.

#### In-depth interview

In-depth-interviews were performed by the principal investigator (RAE). In-depth-interviews were conducted by reading the Arabic version of the Structured Telephone Interviews Questionnaire on Chronic Pain verbatim to gather information about pain, impact on quality of life (including daily activities), treatments used and attitudes about living with chronic pain. Respondents were then invited to answer questions read from an Arabic translation of the S-LANSS. Interviews were terminated after this data was gathered.

### Data analysis and quality control

Data was processed using Statistical Package for Social Sciences (SPSS) version 19.0. Descriptive statistics were used to analyse report demographics, pain location, cause and duration of pain, pain intensity and treatments used. Univariate odds ratios (OR) of respondents who had chronic pain were calculated for each demographic and different groups. When there were more than one group in any category, e.g. age groups, a reference group was chosen in line with those used in previous studies [14, 15].

Multivariate analysis was conducted through the logistic regression function of SPSS with the method chosen as *Enter* with the dependent variable being the answer to the question (for example, “Are you adult aged 18 and older who suffers from pain from an illness or medical condition?”) and the independent variable being sex, age groups, marital and employment status. Mutual adjusted odds ratios were calculated with all independent variables taken into account. Furthermore, a matrix Spearman Correlation was conducted to investigate co-linearity between demographic factors.

Prevalence data was reported as percentage with mean and standard deviation used to summarize data subsets. Chi-square (*X*^*2*^) tests were used to compare proportions and to test for associations between categorical socio-demographic variables. *T*-test or analysis of variance was used to explore the mean differences of normally distributed data when appropriate. Respondents’ views about the interview process were analysed descriptively.

### Role of the funding source

The funding source provided financial support for the first author and had no role in study design, analysis and interpretation of data and in the writing of this manuscript or in the decision to submit it. The funding source provided the permission to collect data and provided two of its employees to help data collection in the first round of interviews.

## Results

### Response rate

Of 2500 telephone numbers that were dialled, 1226 were categorized as unsuccessful calls because the number was not valid (*n* = 356); there was an “out of service” ring tone (*n* = 276); no one replied (*n* = 199); there was an engaged ring tone (*n* = 297); and the number connected to a fax machine (*n* = 98). Of the 1274 telephone calls that were answered 43 respondents did not wish to participate in the screening interview and 19 respondents were not eligible for the study because they declared that they were not Libyan (*n* = 8) or declared that they were younger than 18 years of age (*n* = 11). Thus, 1212 individuals consented to participate in the survey (response rate = 95 · 1 %). Mean ± SD age was 37 · 8 ± 13 · 9 years (women =37 · 7 ± 12 · 5 years, men = 37 · 8 ± 13 · 9 years, range 18 to 72 years) with more women participating than men (women *n* = 662, 54 · 6 %, n = 550, 45 · 4 %, z-test = 3 · 2, *P* =0 · 001). The demographic characteristics of the 1212 individuals who were screened are summarized in Table 1.

**Table 1:** Prevalence of chronic pain, crude odd ratios and odd ratios adjusted for gender and age according to different demographic factors

	N (%) *Reference group	No Pain	Pain	Prevalence of chronic Pain % (95 % CI)	Crude OR (95 % CI)	*P*	Adjusted OR (95 % CI)	*P*
Gender								
Male	550 (45 · 4)*	472	78	14 (11 · 1–16 · 9)				
Female	662 (54 · 6)	502	160	24 (20 · 7–27 · 2)	1 · 9 (1 · 4–2 · 6)	<0 · 001	1 · 9 (1 · 4–2 · 7)	0 · 000
Age(years)								
≤ 30	431 (35 · 6)*	384	47	10 · 9 (8 · 0–13 · 8)				
31–40	373 (30 · 8)	329	44	11 · 8 (8 · 5–15 · 1)	1 · 1 (0 · 7–1 · 7)	0 · 691	0 · 9 (0 · 6–1 · 5)	0 · 706
41–50	183 (15 · 1)	125	58	31 · 7 (25 · 0–38 · 4)	3 · 8 (2 · 4–5 · 8)	<0 · 001	3 · 3 (2 · 1–4 · 5)	0 · 000
51–60	149 (12 · 3)	86	63	42 · 3 (34 · 4–50 · 2)	6 · 0 (3 · 8–9 · 3)	<0 · 001	5 · 1 (3 · 1–8 · 4)	0 · 000
61–70	61 (5 · 0)	47	14	22 · 9 (12 · 4–33 · 4)	2 · 4 (1 · 2–4 · 7)	0 · 009	2 · 4 (1 · 1–5 · 0)	0 · 021
≥ 71	15 (1 · 2)	3	12	80 · 0 (59 · 8–100 · 0)	32 · 6 (8 · 9–120 · 0)	<0 · 001	69 · 0 (14 · 0–339 · 9)	0 · 000
Marital status								
Single	450 (37 · 1)*	397	53	11 · 8 (8 · 8–14 · 8)				
Married	669 (55 · 2)	523	176	26 · 3 (23 · 0–29 · 6)	2 · 5 (1 · 8–3 · 5)	<0 · 001	1 · 3 (0 · 9–2 · 1)	0 · 173
Divorced	36 (3 · 0)	31	5	13 · 9 (2 · 6–25 · 2)	1 · 2 (0 · 4–3 · 2)	0 · 707	0 · 5 (0 · 2–1 · 5)	0 · 214
Widowed	57 (4 · 7)	53	4	7 · 2 (0 · 5–13 · 9)	0 · 6 (0 · 19–1 · 6)	0 · 290	0 · 1 (0 · 0–0 · 4)	0 · 002
Employment status								
Employed full-time	296 (24 · 4)*	250	46	15 · 5 (11 · 4–19 · 6)				
Employed part-time	322 (26 · 6)	275	47	14 · 6 (10 · 7–18 · 5)	0 · 9 (0 · 6–1 · 4)	0 · 743	1 · 2 (0 · 8–2 · 0)	0 · 407
Retired	78 (6 · 4)	53	25	32 · 1 (21 · 7–42 · 5)	2 · 6 (1 · 4–4 · 5)	0 · 001	1 · 2 (0 · 6–2 · 3)	0 · 603
Not employed	317 (26 · 2)	216	101	31 · 9 (26 · 8–37 · 1)	2 · 5 (1 · 7–3 · 8)	<0 · 001	2 · 3 (1 · 4–3 · 5)	0 · 000
Student	144 (11 · 9)	127	17	11 · 8 (6 · 5–17 · 1)	0 · 7 (0 · 4–1 · 3)	0 · 029	1 · 1 (0 · 5–2 · 2)	0 · 817
Temporary out of work	55 (4 · 6)	53	2	3 · 6 (1 · 3–8 · 5)	0 · 2 (0 · 0–0 · 9)	0 · 032	0 · 2 (0 · 0–1 · 1)	0 · 063
Total	1212	974	238	19 · 6 (14 · 6–24 · 6)				

*Reference group

### Prevalence of chronic pain

**Fig. 1:**
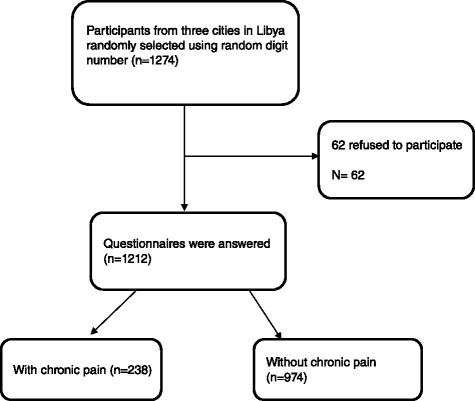
Flow chart of results of recruitment

Of the 1212 individuals who were screened, 238 reported that they experienced pain that had persisted for at least 3 months (Fig. 1). The prevalence of chronic pain across the 3 cities was estimated to be 19 · 6 % (95 % CI 14 · 6 % to 24 · 6 %). The prevalence of chronic pain in Tripoli was 25 · 1 % (95 % CI 21 · 6 % to 28 · 4 %; 158 of 630 respondents), in Benghazi was 13 · 0 % (95 % CI 10 % to 16 %, 63 of 483 respondents) and in Sabha was 17 · 2 % (95 % CI = 9 · 6 % to 24 · 4 %, 17 of 99 respondents). There were statistically significant differences in prevalence between the cities (*X*^2^ = 33 · 7, *P* <0 · 001).

The mean ± SD age of respondents reporting chronic pain was 45 · 5 ± 1 years and significantly higher than those that did not report chronic pain (35 · 9 ± 11 · 9, *t*-test = −9 · 8, *P* <0 · 001). The mean ± SD age of men reporting chronic pain was significantly higher than women (men = 47 · 7 ± 17 · 4 years, women = 44 · 4 ± 13 · 8 years, *t*-test = 3 · 6, *P* < 0 · 001).

**Fig. 2:**
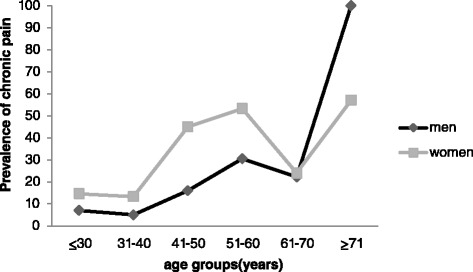
Age-sex specific prevalence (% out of respondents in each age group) of chronic pain in Libya

Prevalence increased across the lifespan from 10 · 9 % (95 % CI 8 · 0-13 · 8) in respondents less than 30 years of age to 80 · 0 % (95 % CI 59 · 8-100 · 0) in respondents greater than 70 years of age, although prevalence for the age category 61–70 years was 22 · 9 % (95 % CI 12 · 4-33 · 4) and lower than the 51–60 year age category (42 · 3 %, 95 % CI 34 · 4-50 · 2) and the 41–50 years age category (31 · 7 %, 95 % CI 25 · 0-38 · 4). Respondents aged 71 years or older were more likely to report chronic pain than other age categories and had a higher odds ratio for reporting chronic pain than respondents less than 30 years (OR = 32 · 6; 95 % CI 8 · 9-120 · 0, *P* <0 · 001, Table 1). Women were more likely to report chronic pain than men (women = 24 · 0 %, 95 % CI 20 · 7- 27 · 2, men = 14 · 0 %, 95 % CI 11 · 1-16 · 9) irrespective of age (*X*^2^ = 1 · 1, *P* < 0 · 298, OR = 1 · 9; 95 % CI 1 · 4-2 · 6) and across all age categories except the age category of 61–70 years (Fig. 2).

The prevalence of chronic pain was higher in respondents that were married (*X*^2^ = 42 · 9, *P* < 0 · 001, OR = 2 · 5; 95 % CI 1 · 8-3 · 5). Widowed respondents were less likely to report chronic pain than other groups (Table 1). Prevalence differed according to employment status (*X*^2^ = 61 · 4, *P* < 0 · 001). The highest proportion of chronic pain was reported by retired and unemployed respondents reporting chronic pain (and the lowest by respondents who were employed, students, or ‘temporarily out of work’ (Table 1). The odds ratio for chronic pain was no different in retired and unemployed respondents than students and ‘temporarily out of work’ (Table 1).

### Prevalence of neuropathic pain

The prevalence of neuropathic pain in respondents reporting chronic pain was 19 · 7 % (95 % CI 14 · 6-24 · 7) with 47 of 238 chronic pain respondents classified as S-LANSS positive with a score of ≥12. Thus, the prevalence of neuropathic pain in the whole sample was 3 · 9 %, (95 % CI 2 · 8 to 5 · 0 %).

### Multivariate analysis

Crude odds ratios found that sex and age were the most significant factors associated with chronic pain. Being married, retired or unemployed also increased the odds ratios of having chronic pain, although being married or retired was no longer a risk factor when other factors were incorporated into the equation of logistic regression and all factors were mutually adjusted (*P* = 0 · 173, *P* = 0 · 603). Adjusted odds ratios showed that unemployment increased the odds of having chronic pain (OR = 2 · 3; 95 % CI 1 · 4-3 · 5) and being widowed decreased the odds of having chronic pain (OR = 0 · 1; 95 % CI 0 · 0-0 · 4). There was a strong correlation between marital status and age groups (*r* = −0 · 4, *P* < 0 · 01) and therefore marital status was removed from the logistic regression model and new adjusted odds ratios were produced (Table 2). Unemployment remained a risk factor of having chronic pain but there were no other significant changes to the odds ratios suggesting that removal of marital status had little effect on the model.

**Table 2:** Logistic regression model of socio-demographic factors significantly associated with chronic pain after removal of Marital Status

	*N* (%) *Reference group	Adjusted OR (95 % CI)	*P*
Gender			
Male	550 (45 · 4)*		
Female	662 (54 · 6)	1 · 9 (1 · 3–2 · 6)	< 0 · 001
Age (years)			
<30	431 (35 · 6)*		
31–40	373 (30 · 8)	0 · 9 (0 · 6–1 · 5)	0 · 784
41–50	183 (15 · 1)	3 · 4 (2 · 1–5 · 3)	< 0 · 001
51–60	149 (12 · 3)	5 · 5 (3 · 4–8 · 5)	< 0 · 001
61–70	61 (5 · 0)	2 · 4 (1 · 2–5 · 0)	0 · 017
>71	15 (1 · 2)	34 · 0 (8 · 4–138 · 4)	< 0 · 001
Employment status			
Employed full-time	296 (24 · 4)*		
Employed part-time	322 (26 · 6)	1 · 2 (0 · 7–1 · 9)	0 · 491
Retired	78 (6 · 4)	1 · 1 (0 · 6–2 · 3)	0 · 578
Not employed	317 (26 · 2)	2 · 3 (1 · 4–3 · 5)	0 · 002
Student	144 (11 · 9)	2 · 0 (1 · 3–3 · 1)	0 · 788
Temporary out of work	55 (4 · 6)	0 · 2 (0 · 0–1 · 0)	0 · 053
Total	1212		

*Reference group

#### Duration and frequency of chronic pain

**Fig. 3:**
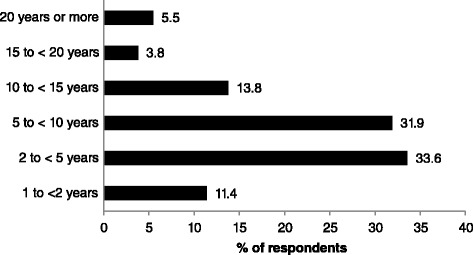
Variations in duration of chronic pain of respondents. Data collected from answers to question 6 “For how long have your suffered from pain due to your illness or medical condition?”

Mean ± SD duration of pain rated on 1–10 numerical rating scale (NRS) was 6 · 5 ± 5 · 7 years (*n* = 238). The majority of respondents with chronic pain reported that they experienced pain that persisted for 2 to 10 years (*n* = 156 (65 · 5 %)), with 13(5 · 5 %) respondents reporting that they had experienced pain that persisted for more than 20 years (Fig. 3). There were no differences in mean ± SD duration of pain rated at an intensity of 5 or more between women and men (women = 6 · 6 ± 5 · 9 years, men = 6 · 3 ± 5 · 3 years, *t* = −0 · 4; *P* = 0 · 693, *t*-test). Of the 238 respondents categorised as having chronic pain, 27 (11 · 3 %) reported experiencing pain ‘At all times’ and 89 (37 · 4 %) reported experiencing pain ‘Daily’. Seventy-five (31 · 5 %) of respondents categorised as having chronic pain reported that they experienced pain ‘Today’ (i.e. on the day of interview), and 90 (37 · 8 %) reported that they experienced pain ‘Not today, but within the past week’.

#### Pain intensity, tolerance to pain, and time course of pain

**Fig. 4:**
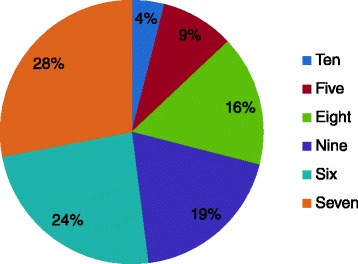
The intensity of the most recent episode of pain for respondents with chronic pain (*n* = 238) in answer to the screening question number 9” Thinking about the last time you experienced pain, please give me a number from 1 to 10 to indicate the intensity of your pain. Please use a 10-point scale where 1 means” no pain at all” and 10 mean” the worst pain imaginable”. All participants with chronic pain have scored their pain 5 or more

The intensity of the most recent episode of pain in the 238 respondents reporting chronic pain was 7 · 2 ± 1 · 3 on a 10-point numerical rating scale (1 = ‘no pain at all’ and 10 = ‘the worst pain imaginable’), and gleaned from the question: “Thinking about the last time you experienced pain, please give me a number from 1 to 10 to indicate the intensity of your pain.” One hundred and forty-six of the 238 (61 · 3 %) respondents reporting chronic pain rated the intensity of their most recent episode of pain between 5 and 7 (i.e. moderate) and 92 (38 · 7 %) rated the intensity of their most recent episode of pain between 8 and 10 (i.e. severe, Fig. 4). The intensity of the most recent episode of pain was rated higher for women compared with men (*t* = −3 · 505, *P* < 0 · 01, *t*-test), with no differences according to age categories (*F* = 1 · 0, *P* = 0 · 386, oneway ANOVA).

**Fig. 5:**
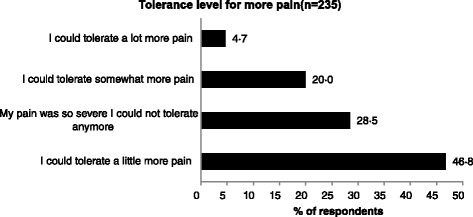
The “tolerance level” when the pain was at its worst for respondents with chronic pain (*n* = 235) in answer to the in-depth interview question,” Thinking about the intensity of your pain when it was at its worst, which of the following statements best describes your tolerance level of this pain”

Sixty-seven (28 · 5 %) respondents with chronic pain reported that “My pain was so severe that I could not tolerate any more, not even a little” (Fig. 5). One hundred and seventy one (71 · 8 %) respondents with chronic pain reported their pain was ‘intermittent’ rather than ‘constant’ to the question, “Would you describe the pain you generally experience as constant, on-going pain that is always there or intermittent pain that comes and goes?”

#### Body location of pain

**Fig. 6:**
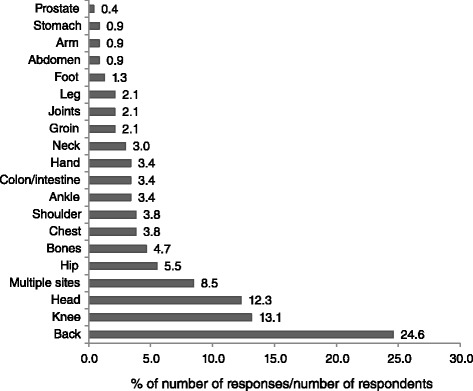
Body location of pain in answer the screening question “Where is your pain located”, and respondents providing answers to the interviewer reading from a list of possible answers, with multiple entries accepted. There were 267 answers (responses) from 236 respondents with 20 respondents providing multiple sites of pain. Percentage responses were calculated by using 236 as the denominator and therefore totals exceed 100 %

Two hundred and sixteen (91 · 5 %) respondents with chronic pain (*n* = 236) reported that they experienced pain in one body site and 16 (6 · 8 %) respondents reported had pain at two sites. The most common sites of pain gleaned from the screening question: “Where is your pain located?” (interviewer read from a list of possible answers, multiple answers accepted) was the ‘back’ (*n* = 58 (24 · 6 %), unspecified (*n* = 30), ‘lower back’ (*n* = 24) and ‘upper back’ (*n* = 4)) and the ‘knee’ (*n* = 31 (13 · 1 %), Fig. 6). There was no difference in the number of sites of pain across age categories (*X*^2^ = 319 · 5; *P* <0 · 001).

#### Causes of pain

**Fig. 7:**
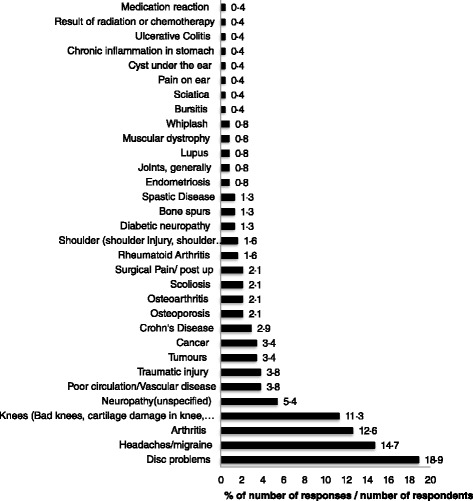
The opinions of respondents on the cause of their pain in answer to the screening question,”Please tell me the illness or medical condition that is the cause of your pain”. Respondents provided answers to the interviewer reading from a list of possible answers, with multiple entries accepted. There were 265 answers (responses) from 229 respondents with 26 respondents providing more than one cause for their pain. Percentage responses were calculated by using 229 as the denominator and therefore totals exceed 100 %

Nine (3 · 8 %) respondents refused to answer the question: “Please tell me the illness or medical condition that is the cause of your pain”. Two hundred and three (88 · 6 %) respondents with chronic pain (*n* = 229) reported one cause for their pain, 22 reported two causes (9 · 6 %). The most commonly reported causes were ‘Disc problems’ (*n* = 45 (19 · 6 %)) and ‘Headaches/migraine’ (*n* = 35 (15 · 3 %), Fig. 7). The proportion of respondents reporting ‘Headaches/migraines’ was higher in women than men (z = 1 · 9; *P* = 0 · 027). In the 203 respondents reporting one cause of pain ‘Disc problems’ (*n* = 42 (18 · 3 %)) and ‘Headaches/migraine’ (*n* = 34 (14 · 8 %)) were most common.

#### Pain descriptors

Respondents provided answers to the question: “What words would you use to describe the pain you generally experience?” (interviewer read from a list of possible answers, multiple answers accepted). There were 63 different answers from 238 respondents with 98 respondents providing more than one descriptor for their pain. The most commonly used words were ‘Aching’ (*n* = 54), ‘Intense’ (*n* = 44), ‘Painful’ (*n* = 35), ‘Annoying’ (*n* = 16), and ‘Excruciating’ (*n* = 24). One hundred-forty respondents (58 · 8 %) used only one word to describe their pain with ‘Aching’ (*n* = 27) being the most commonly used word. Four respondents used ‘Other’ words not listed in the questionnaire which were ‘Unable to move’ (*n* = 2) and ‘Hotness’ (*n* = 2).

#### Impact of pain on daily activities

Respondents were asked to rate their ability to undertake daily activities as ‘just as able’, ‘less able’, or ‘no longer able’ from a list read out by the interviewer. There were 106 (45 %) of respondents who reported that they were ‘Just as able’ to participate in all activities read from the list. Of the 133 (55 %) respondents that reported pain had reduced their ability to undertake daily activities the greatest impact was on walking, lifting exercising and sleeping. Forty three (18 · 1 %) respondents reported that they were ‘No longer able’ to undertake ‘Lifting’ and 44 (18 · 5 %) were ‘No longer able’ to undertake ‘Exercising’.

#### Impact of pain on employment and emotional status

Only 46 (19 · 3 %) respondents reporting chronic pain (*n* = 238) were in full time employment and 64 (27 · 0 %) were in part-time employment or were students. Thirty five respondents reported that pain had impacted on their employment status and 29 reported that they had to change their job responsibilities and 6 reported losing their job or having to change their job. Respondents who were students or in full-time or part-time employment reported that they had lost 10 · 2 ± 5 · 2 days from work (mean ± SD) over the previous 6 months due to pain. Thirteen of 221(5 · 8 %) respondents reported that they had been diagnosed with depression because of their pain.

#### Pain practitioners

**Fig. 8:**
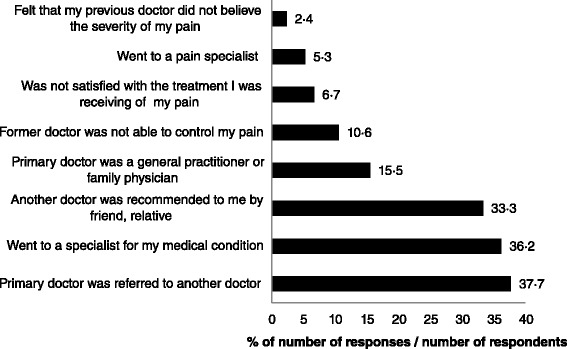
Reasons why respondents with chronic pain see more than one doctor in answer to the question,”Please tell me your reasons for seeing more than one doctor for pain treatment?” with multiple entries accepted. Interviewers did not read from a list but categorised responses against a list on the questionnaire and probed once for ‘what other reasons’. Percentage responses were calculated by using the 208 respondents who had seen more than one doctor as the denominator and therefore totals exceed 100 %

Ninety-seven (40 · 8 %) respondents with chronic pain reported that they had not visited their doctor about their pain in the previous six months. Of the 141 respondents who had visited their doctor in the previous 6 months, 87 (61 · 7 %) reported visiting their doctor more than once, with a mean ± SD 2 · 6 ± 1 · 2 visits over the 6 month period. Nineteen respondents with chronic pain reported that they had consulted only one doctor and 199 had consulted more than one doctors. The main reason for visiting more than one doctor was referral by another doctor or a recommendation by a friend or a family member (Fig. 8).

One hundred and thirty eight (57 · 9 %) respondents had been under the care of their current doctor for management of their pain for 3 months to 10 years. One hundred and three (43 · 3 %) respondents were ‘somewhat satisfied’ with the doctor who currently treating their pain, 71 (29 · 8 %) were ‘very satisfied’ and 21 (8 · 8 %) were ‘extremely satisfied’. Twenty-seven (11 · 4 %) respondents were ‘not satisfied’ or ‘not at all satisfied’ with the main reasons being ‘Has not helped me/has not relieved my pain’ or because they ‘Do not see the same doctor each time’. One hundred and seventy seven (74 · 4 %) respondents reported being uncomfortable discussing their pain with their doctor and preferred to discuss their pain with family members and/or friends.

#### Assessment of pain by doctors

One hundred seventy seven (74 · 4 %) respondents reported that their doctor determines how much pain they were in ‘during every visit’. Twenty (8 · 4 %) respondents reported that their doctor ‘Never’ assessed their pain during a consultation. In answer to the question: “How does the doctor who generally treats your pain determine how much pain you are in?” 157 (66 · 0 %) respondents reported that ‘I tell him/her’. Only 1 respondent reported that their doctor used a pain scale to determine how much pain they were in.

#### Treatment

Only, 71 (29 · 8 %) of respondents reported that their pain was being adequately controlled. Thirty seven (15 · 5 %) respondents reported that they had achieved pain control between 6 months to one year from the time when they first experienced pain with 8 (3 · 4 %) respondents reporting that they had been in pain from 5 to 7 years before they received effective treatment. One hundred and forty six (61 · 3 %) respondents reported that they were not ‘currently being treated’ for their pain with the most common reasons being ‘Do not want surgery’, ‘Can manage pain on own’ and ‘Treatment has not helped’.

One hundred and eighty three (76 · 2 %) respondents reported that they had taken prescription medicine for their pain. Forty eight respondents reported that they never taken prescription medication, of which 14 reported that they could ‘Manage/live with the pain’, 14 reported that pain medication was ‘Never prescribed’, and 9 reported that ‘Pain was not bad enough’. One hundred and five (44 %) respondents reported that they had stopped taking prescription medicine with reasons including ‘Manage/live with the pain’ and ‘Ran out of medication’.

One hundred and three of 156 respondents to the question: “How many different kind of prescription pain medicines have you ever taken for your pain?” reported that they had taken one to two types of prescription medications and 53 respondents reported that they had taken three to eleven types of prescription medications. Forty two respondents reported that their doctor had ‘switched prescription pain medicines’ or ‘prescribed more than one medicine for the same pain’. The most common reason reported for switching pain medication was ‘Pain became worse’. Only 69 respondents were able to name the drug that they were currently being prescribed and only three prescription medicines were named (‘Voltarol’, ‘Ibuprofen’ and ‘Paracetamol’). No respondents reported that their current prescription pain medication was ‘Completely effective’ while 24 reported that they were ‘very effective’.

Ninety nine (41 · 6 %) respondents reported that they had taken non-prescription oral pain medicine in the last six months and 87 of these respondents reported that they had taken one or two non-prescription oral pain medications. The most commonly reported non-prescription oral pain medicines were non-steroidal anti-inflammatory medication (*n* = 55, ‘Voltarol’, ‘Ibuprofen’, ‘Aspirin’) and paracetamol (*n* = 27). Respondents reported that their non-prescription medicines were ‘Not very effective’ or ‘Not at all effective’ (*n* = 19), ‘Somewhat effective’ (*n* = 67), and ‘Very effective’ or ‘Completely effective’ (*n* = 13).

Two hundred and seven (87 %) respondents reported that they had used non-drug methods to treat their pain and the majority of these reported that they used only one non-drug treatment (*n* = 116). Physical therapy, massage and herbal supplements were reported to be the most commonly used non-drug methods to treat pain. Forty six respondents reported that the methods were ‘Extremely successful’, 125 respondents ‘Somewhat successful’, 19 respondents ‘Very successful’ and 17 respondents ‘Not very successful’.

One hundred and one (42 · 4 %) respondents reported that they had been ‘treated for pain’ or ‘seen a doctor in another country’, with ‘Better access [to pain treatment]’ (*n* = 49) and ‘Better reimbursement in the other country’ (*n* = 36) being the most common reasons. Egypt and Tunisia were the most common countries used in addition to Jordan and England.

#### Attitudes and beliefs about pain and its treatment

The interviewer read 33 statements describing how people experiencing pain think and feel about their pain and the respondent was asked to reflect on their own pain and to ‘completely’ or ‘somewhat’ agree or disagree with each statement using a 5-point Likert scale. This task was toward the end of the interview and only 71 (29 · 8 %) respondents agreed to rate the statements. Pain clearly impacted on daily activities and a sense of wellbeing. Six respondents replied (8 · 5 %) ‘agreed completely’ and 27 (38 %) ‘agreed somewhat’ that ‘I cannot function normally’. Twenty nine respondents (40 · 8 %) ‘agreed completely’ or ‘agreed somewhat’ with the statement ‘Some days the pain is so bad, I want to die’. Six of the 71 respondents (8 · 2 %) respondents ‘agreed completely’ and 17 (23 · 3 %) ‘agreed somewhat’ that ‘I worry about what people would think if they knew I take pain medicine’ and 18 (25 · 4 %) respondents agreed completely that ‘I would spend all my money on pain treatment if I knew it would work’. Fifteen of the 71 (21 · 1 %) respondents completely agreed that ‘My doctor would rather treat my illness than my pain’ and 11 (15 %) reported that ‘I think my doctor does not know how to control my pain’.

## Discussion

This is the first study to estimate the prevalence of chronic pain in Libya. It was found that the prevalence in adult general population was 19 · 6 %. This is lower than the overall weighted mean prevalence worldwide that was found to be 30 · 3 % ± 11 · 7 % by Elzahaf and colleagues [7] and lower than the previous worldwide estimates by Smith & Torrance [16], which was 22 · 9 % (95 % CI 22 · 7 %- 23 · 2 %), and Harstall & Ospina [17], which was 35 · 5 %. Perhaps most importantly, the Libyan estimate was lower than the weighted mean prevalence in the Middle East and North Africa, which was found to be 28 · 0 % ± 9 · 2 [7, 12] and lower than estimates of individual counties in the region (e.g. Lebanon (26 · 2 % [8]) and Turkey (28 · 9 % [9]). It is, however, comparable to a recent telephone survey of 16 regions of Morocco that estimated prevalence of reported chronic daily pain for more than 3 months in the general population as 21 % (95 % CI: 19 · 9-22 · 2), and higher in women and individuals older than 60 [18].

The Libyan estimate was also comparable to the overall prevalence of chronic pain in Europe (19 % [10]) and Canada (18 · 9 % [19]) which gathered data using the same Structured Telephone Interviews Questionnaire on Chronic Pain as used in this Libyan study. Libya is classified as a developing country with an HDI of 0 · 784 (2013) and the Libyan estimate of prevalence was lower than many estimates from countries with similar HDIs <0 · 9 including Colombia (27 · 3 %, HDI = 0 · 807, [8]), Brazil (30 · 8 %, HDI = 0 · 790, [9]), Mexico (24 · 1 %, HDI = 0 · 854, [8]), Chile (33 %, HDI = 0 · 719, [9]), South Africa (48 · 3 %, HDI = 0 · 683, [8]), Nigeria (30 · 4 %, although one survey reported 5 · 5 %, HDI = 0 · 511, [8, 9]), and India (19 %, HDI = 0 · 566, [9]).

This survey estimated that the prevalence of neuropathic pain in the general adult population of Libya using the S-LANSS questionnaire was 3 · 9 % (95 % CI = 1 · 4 % to 6 · 4 %). This estimate is similar to surveys in Austria (3 · 3 %) [20] and Spain (3 · 3 %) [21] but lower than the UK (8 · 2 %, [22]), Canada (17 · 9 %, [23]) and France (6 · 9 %, [24]). In Western Europe, direct medical costs associated with neuropathic pain was found to be approximately twice as high as non-neuropathic pain [25]. No such direct medical costing was attempted in this study in Libya but anecdotal evidence suggests that a high number of patients with untreated pain seek pain management services and advice from outside the country, which is likely to be expensive. Further research is needed on the aetiology of neuropathic pain in Libya.

The main causes of chronic pain in Libya were back pain, osteoarthritis, rheumatoid arthritis and headaches, and similar to that reported by Breivik and colleagues [10] in the pan-European survey. Breivik and colleagues [10] found that at least 15 % of chronic pain conditions resulted from trauma or surgery although only 5 · 9 % of chronic pain conditions were in this category from our Libyan survey. We suspect that this was because of fewer surgical procedures performed in Libya compared with countries in Europe because of many Libyan patients undergoing surgeries abroad [26].

It was found that in Libya women were more likely to have chronic pain than men, a finding consistent with previous surveys regardless of survey method used [10, 15, 22, 24, 27, 28]. The odds ratio was 1 · 9 and higher than recent surveys in Ireland (1 · 0, [14]), Hong Kong (1 · 5, [15]), and lower than a recent survey in Portugal (2 · 3 [29]). Research evidence supports sex-based differences in pain experience [30–32] including a higher prevalence and severity of chronic pain in women than men [33]. Tashani and colleagues [34] have found that Libyan women have greater sensitivity to experimentally induced pain than Libyan men and that there is a strong correlation between gender role and pain sensitivity response [35]. The present findings provide more evidence that this trend is universal.

Our survey found that in Libya the prevalence of chronic pain increased with age and this is consistent with previous studies [28, 29, 36, 37]. In Libya, the prevalence of chronic pain was highest in the oldest age group (>70 years), although the sample size in this age category was small. We suspect that the higher prevalence of chronic pain in old age was related to an increased possibility of having a chronic progressive disease [38].

The demographic factors which were associated with an increased likelihood of having chronic pain in Libya were being a woman, of advanced age and unemployed. In Libya women are more likely to be unemployed than men so there is co-linearity between unemployment and being a woman, and therefore being unemployed in Libya is probably not a strong risk factor for having chronic pain. It is also possible that, at least for a proportion of the population, being out of employment could be the result of having chronic pain. In Libya, sexual relationships outside of wedlock are prohibited and socially unacceptable and therefore most women are married. We found that the significance of being married as a risk factor for chronic pain disappeared when adjusted for sex and age.

A large proportion of Libyan adults with chronic pain reported taking prescription medicine to treat their pain with NSAIDs being the most common class of analgesic medication used. This finding is consistent with other surveys in other regions of the world [10, 39–44]. In Libya, the use of opioid analgesics is only available via prescription [45]. Soyannwo [46] reported that in resource limited countries there is a fear of opioid abuse, dependence, and addiction. For example, a study conducted in Nepal found that opioid analgesics were not used by chronic pain patients [39]. Legal restrictions, policy, knowledge and attitudes continue to contribute to the unavailability of opioid analgesics in the Middle East and North Africa. There is a need for education and training programmes in Libya about the use of opioids in treating chronic pain patients.

One unexpected finding from the present study was the similarity of non-pharmacological approaches to reduce pain with that found in the pan-European survey, with few ‘traditional remedies’ reported to be used by Libyan respondents. The Libyan sample was taken from large cities where individuals had access to modern health care services, similar to those found in resource rich countries. Non pharmacological approaches for the management of pain tend to be delivered by physiotherapists within rehabilitation services in a manner similar to that seen in European countries. Some of the physiotherapists are qualified to BSc level and they work within multi-disciplinary teams in a variety of medical departments in public hospitals. Nevertheless, equipment, facilities and time spent with patients is often inadequate due to limited resources. In recent years the need for physiotherapy and rehabilitation services has increased as a result of the conflict and the number of private clinics is on the rise. A minority of respondents reported that they had tried to treat their pain with olive oil (orally or topically), cupping (Hjama), burning skin (Kai) and local herbal concoctions (Kmra).

### Limitations

Estimation of chronic pain in the general population relies on recall of pain status during a defined period of time and is susceptible to recall bias and therefore inferences are made with caution. Some questions relied on respondents to self-report their condition and the accuracy of these reports is likely to be poor, especially for specific diagnoses such as “disc problems” which are challenging to medical specialists even with in-depth clinical assessments and the most sophisticated of technology. Moreover, data was collected from a cross-sectional survey and in some instances it was unclear whether the respondents were describing pain before, during or after treatment. Elderly respondents who had difficulty communicating were helped by other members of the household, which may have influenced their responses. The questionnaire used to collect data was designed for use in European countries and was translated and culturally adapted for use in Libya. However, some questions or words, which were designed for European patients, could have being misunderstood by Libyans respondents in the same way.

The most obvious direction for future research is to conduct a post-conflict follow-up study as this would provide valuable information about trends in the prevalence of chronic pain in regions affected by conflict. This would enable policy makers to undertake up-to-date analyses of pain management needs. A future study could also adjust for factors such as smoking and body mass index.

## Conclusion

The prevalence of chronic pain ≥ 3 months in the general adult population of 3 major cities in Libya was estimated to be 19 · 6 % (95 % CI = 14 · 6 % to 24 · 6 %) with the prevalence of neuropathic pain being 3 · 9 %, (95 % CI: 2 · 8 to 5 · 0 %). This suggests that chronic pain is a public health problem in Libya with a prevalence comparable to Europe. Risk factors were being a woman, advanced age and unemployment. There is a need for improved pain management policies in Libya to ensure that patients with chronic pain receive effective treatment.
